# Studies of Release Activity in DRGs of Rats with Spared Nerve Injury (SNI) Using YO-PRO-1

**DOI:** 10.17912/micropub.biology.001619

**Published:** 2025-07-11

**Authors:** Yanping Gu, Guangwen Li, Li-Yen Mae Huang

**Affiliations:** 1 Neurobiology, University of Texas Medical Branch

## Abstract

We report a new method for examining changes in membrane permeability in dorsal root ganglion (DRG) cells under peripheral nerve injury conditions. By intravenously injecting YO-PRO-1, a monomeric cyanine nucleic acid stain, into rats with spared nerve injury (SNI), we observed the uptake of YO-PRO-1 in injured neurons and surrounding non-neuronal cells. This approach provides a tool for detecting in vivo DRG cell release activity in intact animals.

**Figure 1. Uptake of YO-PRO-1 in rat DRGs after spared nerve injury (SNI):**
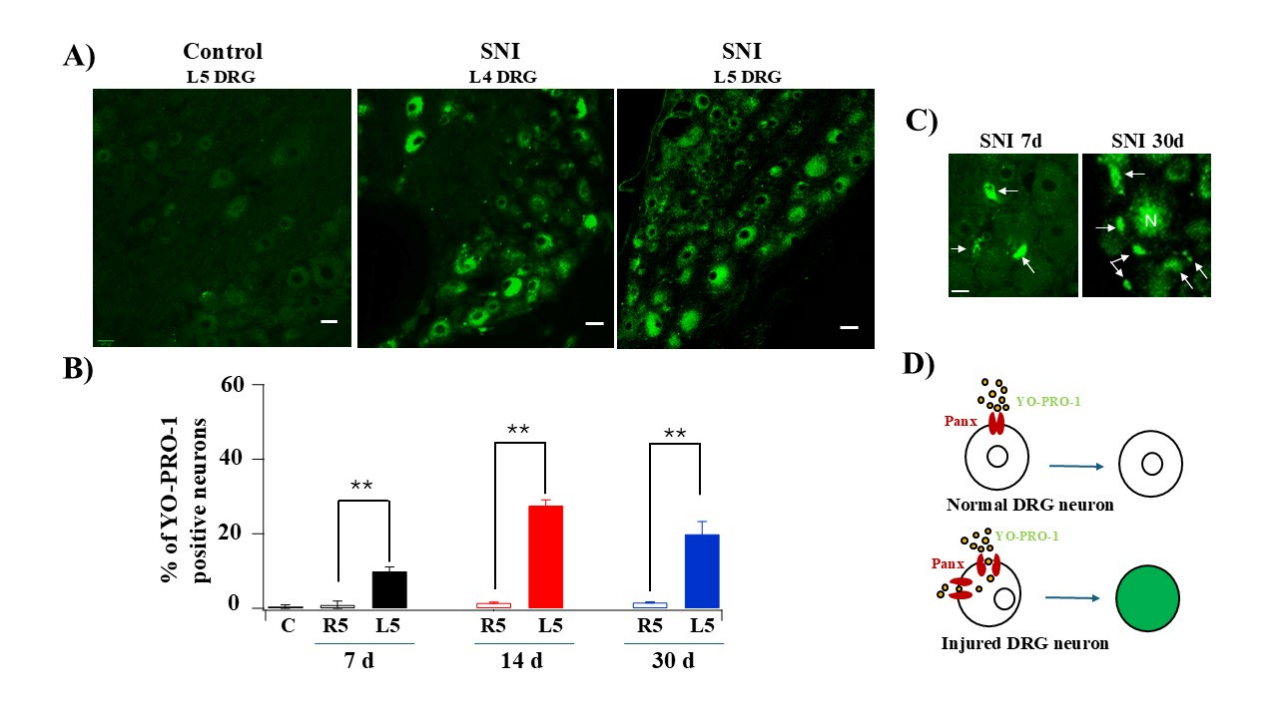
A) YO-PRO-1 (green fluorescence) labeling in DRGs isolated from control and SNI rats. Left panel: L5 dorsal root ganglion (DRG) from a control rat. Middle panel: L4 DRG from an SNI rat. Right panel: L5 DRG from an SNI rat. Scale bars: 25 µm (left panel); 30 µm (middle and right panels). B) Quantification of YO-PRO-1 uptake in DRG neurons from control (C) rats and at 7 days (7d), 14 days (14d), and 30 days (30d) post-SNI. R5: Right (uninjured) L5 DRG; L5: Left (injured) L5 DRG. N=4. **P < 0.01 (Student’s t-test). C) YO-PRO-1 labeling of non-neuronal cells (arrows) in DRGs isolated at 7 and 30 days post-SNI. "N" indicates neurons. Scale bar: 15 µm. D) Schematic illustration of the proposed mechanism of YO-PRO-1 uptake by DRG neurons under SNI conditions.

## Description

Dorsal root ganglion (DRG) neurons play essential roles in the detection, transmission, and modulation of pain signals (Basbaum et al. 2009). Following peripheral nerve injury, not only the distal axon but also the somata of injured neurons undergo significant molecular and functional changes. Increased excitability of these injured DRG neurons is considered a major contributor to neuropathic pain. It is widely recognized that both neurons and non-neuronal cells in DRGs release substances to enhance DRG neuronal activity (Gu et al., 2010; Huang et al 2013). Due to the anatomical complexity of the DRG and the lack of tools for simultaneously targeting multiple cell types in vivo, most studies investigating DRG release activity—the release of substances from DRG cell bodies—have been conducted in vitro (Gebhardt et al., 2020; Qiao et al., 2016). However, direct evidence of in vivo changes in release activity in DRG neurons and glial cells, particularly following nerve injury, remains limited. Changes in membrane permeability can reflect this release activity. YO-PRO-1 uptake has been used as an indicator of membrane permeability changes. Therefore, measuring changes in YO-PRO-1 uptake in DRG cells can reflect changes in their release activity. YO-PRO-1 is a nucleic acid-binding fluorescent dye that only emits green fluorescence upon entering cells and binding to intracellular DNA or RNA. With a molecular weight of ~630 Da, YO-PRO-1 does not cross intact cell membranes but can enter cells with compromised membrane integrity, making it a reliable indicator of membrane permeability changes. While YO-PRO-1 is commonly used to stain cultured cells, its application in live or fresh tissue samples is rare (Chagnon et al. 2010). In this study, we intravenously administered YO-PRO-1 in rats following peripheral nerve injury to examine the permeability of DRG neurons and non-neuronal cells. This method enables simultaneous assessment of both functional (e.g., uptake activity) and structural (e.g., cell localization and morphology) changes, providing direct in vivo evidence of release activity in DRG cells. Figure 1 shows YO-PRO-1 uptake in DRGs isolated from control and SNI rats. In DRGs from control animals, no YO-PRO-1 staining was observed in neurons. In contrast, in DRGs from SNI rats at 14 days post-injury, approximately 25–30% of neurons in the L4 and L5 DRGs were YO-PRO-1-positive ( Fig. 1A, B). The number of YO-PRO-1-positive neurons increased over time post-injury ( Fig. 1B ). At 7 days post-SNI, 10–15% of neurons were positive, rising to ~30% by day 14. Even at 30 days after SNI, 20–25% of neurons continued to show YO-PRO-1 uptake, indicating persistent membrane permeability changes in injured DRG neurons. In addition to neurons, some non-neuronal cells in the DRGs of SNI rats also took up YO-PRO-1 ( Fig. 1C ). These YO-PRO-1-positive cells, first observed 7 days after injury, exhibited kidney- or bean-shaped nuclei—characteristic of macrophages or satellite glial cells (SGCs)—and were rarely present in DRGs from control or contralateral sides. Their numbers increased over time following injury. These findings suggest that, like neurons, non-neuronal cells may also undergo permeability changes and participate in substance release under injury conditions. Several types of non-neuronal cells reside within the DRG, including macrophages and SGCs. To identify which cell types take up YO-PRO-1, we plan to perform immunostaining with cell-specific markers in future studies. Furthermore, we propose using YO-PRO-1 as a functional tool to investigate neuron–glia interactions during peripheral nerve injury. How does YO-PRO-1 enter DRG neurons? It has been reported that pannexin channels—large-pore membrane channels—contribute to neuropathic pain in DRG neurons (Zhang et al. 2015). We propose a model ( Fig. 1D ) in which YO-PRO-1 enters DRG cells through activated pannexin channels. Under normal conditions, these channels remain closed, preventing dye entry. Following injury, pannexin channels open, allowing YO-PRO-1 to pass through and bind to intracellular nucleic acids, resulting in detectable fluorescence. In non-neuronal DRG cells, YO-PRO-1 uptake may occur via P2X7 receptors (P2X7R), which are known to be upregulated after peripheral nerve injury. This is supported by a report from Patrice Rat and colleagues (Rat et al. 2017), which showed that P2X7R activation can facilitate YO-PRO-1 entry into cultured cells.

## Methods

**Animals** Adult male Sprague-Dawley rats (200–250 g) were purchased from Charles River. Animals were housed under a 12 h light/dark cycle at 25 ± 1°C with food and water available ad libitum. All protocols were approved by the Institutional Animal Care and Use Committee (IACUC) at the University of Texas Medical Branch and followed NIH and IASP guidelines. **Nerve Injury Model** The spared nerve injury (SNI) model was performed as described by Woolf group (Decosterd and Woolf 2000). Under isoflurane (5%) anesthesia, the sciatic nerve and its branches were exposed. The common peroneal and tibial nerves were ligated and transected, with a 2 mm segment excised to prevent regeneration. Sham-operated animals underwent the same procedure without nerve ligation or transection. **YO-PRO-1 Administration** YO-PRO-1 iodide (ThermoFisher, Cat. #Y3581) was used. Rats were anesthetized with pentobarbital (50 mg/kg, i.p.), and YO-PRO-1 (5 µM in 0.2 ml saline) was administered via tail vein injection at 0, 7-, 14-, or 30-days post-injury. One-hour post-injection, rats were perfused with 4% paraformaldehyde. L4 and L5 DRGs were harvested, post-fixed, embedded in OCT, and sectioned for analysis. **Fluorescence Imaging and Data Analysis** YO-PRO-1 fluorescence was imaged using a Nikon MP1 multiphoton microscope (excitation at 488 nm). Z-stack images were acquired due to the 3D structure of DRGs. Quantitative analysis was conducted on 4–5 animals per group. Data are presented as mean ± SEM. Student’s t-test (paired or unpaired) was used for comparisons. A p-value < 0.05 was considered statistically significant.
